# A hyperspectral imaging framework integrating band selection and deep learning for beverage stain classification in forensic analysis

**DOI:** 10.1038/s41598-026-49928-8

**Published:** 2026-05-16

**Authors:** Jitendra Shit, Partha Pratim Roy, V. M. Manikandan

**Affiliations:** 1 Department of Computer Science and Engineering, SRM University-AP, Amaravati, 522240 Andhra Pradesh India; 2 Centre for Interdisciplinary Research, SRM University-AP, Amaravati, 522240 Andhra Pradesh India; 3 Department of Computer Science and Engineering, IIT (ISM) Dhanbad, Jharkhand, 826004 India

**Keywords:** Forensic hyperspectral imaging, Crime scene analysis, Spectral band selection, Deep learning, Beverage stain classification, Trace evidence detection., Engineering, Mathematics and computing, Optics and photonics

## Abstract

The technique of Hyperspectral Imaging (HSI) is significant in the field of non-destructive forensic crime scene investigation, as it allows for the identification of minor spectral changes over a broad range of wavelengths. In the present study, the spectral characteristics of nine beverage stains, such as Papaya, Coffee, Pomegranate, Orange, Tea, Wine, Whisky, Rum, and Brandy, were studied by simulating a controlled environment for a mock crime scene. The hyperspectral images were collected by an HSI system with 204 spectral bands in the visible and near-infrared (VNIR) range. To eliminate spectral redundancy, the ANOVA-based feature selection technique was implemented, which selected 162 spectral bands. These spectral characteristics were employed to train four architectures of deep learning for the classification of the beverage stains, which were implemented as Multi-Layer Perceptron (MLP), one-dimensional Convolutional Neural Network (1D-CNN), Long Short-Term Memory (LSTM), and CNN-LSTM. The training process was carried out using standardized spectral data with adaptive learning rates and early stopping for stable convergence. The strength of the models was evaluated through five-fold cross-validation on stratified data. The experimental results demonstrate that the MLP model attained the greatest classification accuracy of 95.58%. The results show that there is great potential for combining hyperspectral imaging with deep learning for non-destructive stain identification in forensics.

## Introduction

Forensic science is important in any criminal case since it identifies, analyzes, and interprets trace evidence that is unnoticeable to the human eye. Very recently, this approach has been adopted by forensic scientists. HSI can identify hundreds of different wavelengths all at once without changing the evidence in any way. This is clearly what ordinary cameras cannot do. Each pixel in an HSI image has a unique “signature” based on how light reflects off the substance. This reveals chemical and optical changes which standard cameras cannot identify. HSI detects all the stains, residues, fibers, and other small traces seen in crime scenes. Standard methods for stain detection using a microscope, chemical tests, or even spectrophotometry are not always reliable. They are often time-consuming, may damage the sample, and have problems handling insufficient or poor-quality evidence. These difficulties do not concern HSI. It makes no contact, causes no harm, and protects the evidence. However, HSI produces a tremendous amount of data, most of which are either redundant or highly correlated with each other across the spectral bands. If this redundancy is not properly managed, then the processing becomes computationally inefficient and the overall classification performance degrades^1^. Hence, dimensionality reduction or spectral band-selection procedures are critical for enhancing both efficiency and discriminative ability. In this regard, the ANOVA F-test^2^ proves to be one of the best methods due to its ability to identify the wavelengths that yield maximum separation between different classes of samples^3^.

Although there have been studies on the application of HSI in the analysis of various forensic materials such as blood, inks, and fibers, there have been few studies on the analysis of beverage stains, which are common in forensic practice, such as document evidence or contaminated materials. Beverage stains show complex-spectral variability, depending on composition, substrate absorption, and time-dependent chemical changes (aging), which makes automated classification particularly difficult. To our knowledge, no benchmark dataset exists and no standardized evaluation framework exist for hyperspectral beverage stain analysis, and only a few related studies have been conducted so far, which emphasizes the exploratory and original character of this work^4–6^. This study seeks to address this deficiency research by proposing a framework based on deep learning for hyperspectral images, which can be used for classifying nine different types of beverage stains found on absorbent tissue substrates. It proposes the integration of the ANOVA based spectral band selection technique with multiple deep learning architecture, including MLP, 1D-CNN, LSTM, and hybrid CNN-LSTM architecture, to identify the most effective model for classifying beverage stains. Each architecture is assessed using precision, recall, F1-score, and overall accuracy metrics in order to comprehensively assess classification performance. The dataset contains nine beverage classes, namely Papaya, Coffee, Pomegranate, Orange, Tea, Wine, Whisky, Rum, and Brandy, acquired under similar controlled lighting and substrate conditions.

The objective of this study is to assess several deep learning architectures to establish the most robust framework for hyperspectral stain analysis and to establish a foundation for broader forensic applications such as biological fluid analysis, residue detection, document authentication, and material identification^7,8^. The proposed approach will support developing an intelligent, non-destructive, and automated forensic analytic system that enhances accuracy, reproducibility, and supporting data-driven decision-making in forensic investigations.

The main contributions of this research are as follows:

- A specifically designed hyperspectral image dataset is used, comprising nine different classes of beverage stains, namely Papaya, Coffee, Pomegranate, Orange, Tea, Wine, Whisky, Rum, and Brandy.
- A framework utilizing ANOVA for spectral band selection is given to identify the optimal wavelengths for distinguishing beverage stains. The proposed method diminishes the dimensionality of the 204-band hyperspectral data to an optimal selection of 162 bands.
- Four deep learning models-MLP, 1D-CNN, LSTM, and CNN-LSTM-are compared for the categorization of hyperspectral beverage stains.

The Structuring of the study is as follows: Section Related work presents an overview of the relevant research about HSI for forensic science and material analysis. Section Materials and methods introduces materials and methods, including hyperspectral imaging, sample preparation, feature extraction, band selection, then model building. Section Discussion shows and analyzes the experiment results as well as model comparison. Ultimately, Section Conclusion and future work concludes this article along with examines prospective avenues for further research.

## Related work

HSI has been a game-changer in material analysis. It allows us to detect and classify all types of materials simply by viewing their unique spectral signatures. From hospitals to farms, textile labs, and even crime scenes-the application are extensive. Doctors utilize HSI to find out where exactly a tumor ends and to visualize veins beneath the skin with unprecedented accuracy^9,10^. Agricultural and food scientists utilize deep learning models such as autoencoders and CNN to detect defective produce or contamination in foods without contact^11,12^. Forensic experts use 1D-CNN and traditional machine learning approaches to effectively discriminate various fibers and inks in textiles and documents^13,14^ (Table 1).

**Table 1:** Summary of related works in HSI applications.

Year	Authors & Ref.	Application Domain	Methodology/Model Used	Key Findings
2017	Li et al.^15^	Hyperspectral image classification	3D-CNN for spectral–spatial classification	Demonstrated that 3D CNN proficiently acquires both spectral and spatial characteristics to enhance classification accuracy.
2019	Halicek et al.^9^	Tissue classification	CNN with spectral–spatial analysis	Demonstrated HSI’s potential for identifying biological tissues and tumor margins with high spectral accuracy.
2021	Khan et al.^16^	Medical hyperspectral imaging	Analysis of deep learning trends in HSI	Summarized the most advanced deep learning methods for analyzing medical hyperspectral images and the problems they face.
2021	Pu et al.^17^	HSI classification	Attention-driven CNN with multi-level feature learning	Proposed attention-guided CNN to enhance spectral–spatial feature extraction and make classification more accurate.
2021	EL Abady et al.^14^	Document forgery detection	HSI combined with ML classifiers	Demonstrated HSI’s capability in identifying document forgery using spectral differences in inks and paper.
2022	Huang et al.^13^	Textile fiber identification	1D CNN with spectral feature extraction	Created a model based on 1D-CNN for classifying textile fibers without damaging them using HSI data.
2022	Liu et al.^11^	Food anomaly detection	Autoencoder and self-supervised classifier	Introduced a joint optimization framework for anomaly detection in strawberries using HSI.
2022	Manis et al.^18^	Forensic biological fluid analysis	NIR-HSI with multivariate regression	Utilized NIR-HSI with regression models for non-destructive age assessment of biological fluid stains.
2022	Pallocci et al.^19^	Forensic applications review	Review of HSI in forensic analysis	Reviewed HSI use for detecting blood, fingerprints, and documents, emphasizing non-destructive forensic applications.
2023	Basile et al.^12^	Food and beverage analysis	Spectroscopy and HSI review	Provided a comprehensive review on the use of spectroscopy for non-destructive analysis of plant-based beverages.
2023	Hamza et al.^10^	Biomedical visualization	Three-wavelength index imaging with HSI	Demonstrated visualization of subcutaneous blood vessels using hyperspectral imaging.
2025	Kamarul & Annisa et al.^20^	Hyperspectral image classification	Hybrid CNN–BiLSTM network	Combined CNN and BiLSTM to capture both spectral and sequential dependencies, enhancing classification accuracy.

However, research on beverage stain analysis using hyperspectral imaging is still limited. Beverage stains, which show up all the time in crime scenes, get ignored. A couple of exceptions are^4^: showed that HSI can differentiate between coffee and tea stains, but only using basic spectral distance calculations, without machine learning^15^. developed a spectral-spatial framework for the categorization of images with hyperspectral properties with the three-dimensional Convolutional Neural Network (3D-CNN), which considers spatial and spectral information of a hyperspectral picture to enhance classification accuracy. However, the method depends on the analysis of spatial and spectral data for remote sensing images, whereas the regression-based methods for the aging of biological fluids do not consider the more complex beverage stain compositions that occur in real-world crime scenes. Currently, there is a lack of both a benchmark dataset and a standardized evaluation protocol for hyperspectral beverage-stain characterization, and a minimal number of studies have examined this area. All these aspects underpin both the methodological gap in the field and the exploratory, pioneering nature of this present research. Deep learning has driven hyperspectral classification forward at an incredible pace^21,22^. CNNs are very good at capturing local spectral and spatial patterns^17,19^. Hybrid models that combine CNNs with LSTMs^20^ go one step further, tracking both the spatial layout and changes in spectral data across different wavelengths. But the vast majority of these algorithms are trained and tested on clean, standardized datasets - such as Indian Pines or Pavia University, rather than unexpected materials seen in real forensic investigations. Moreover, it is still not clear how reliable these models are when it comes to explaining decisions and applying them to real-life forensic situations.

That’s where this study comes in. The present work undertakes the classification of beverage stains based on a combined approach integrating the statistical band selection methodology with an ANOVA component for interpretability, along with deep learning methods for enhanced predictive capability. Such a combination provides better performance with improved insight into the discriminative spectral features. Beyond strengthening the classification accuracy, the approach extracts meaningful spectral shifts related to the underlying chemistry of the stains and temporal evolution.

## Materials and methods

The following section explains how beverage stains can be analyzed by HSI and deep learning. The various steps in the methodology include hyperspectral image acquisition, cleaning of data, extraction of features, selection of features, training of models, and evaluation of performance. Each of these steps is necessary to group the various visually similar stains into the correct category based on the spectral signature. These processes make sure that small spectral differences between stains are recorded accurately so that they can be classified appropriately. These differences may not be easy to see with the naked eye.

### HSI and data acquisition

**Fig. 1:**
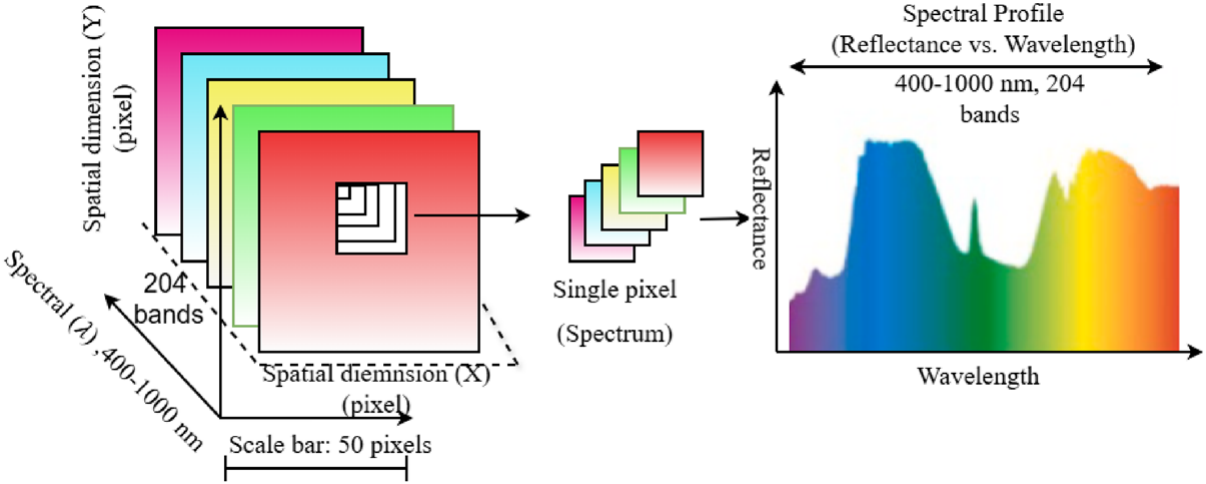
Hyperspectral data cube and spectrum.

The hyperspectral pictures were acquired using the Specim IQ Camera (Specim Ltd.)^23^. The Specim IQ Camera captures images in 204 spectral bands, extending from 400 nm to 1000 nm. The spectral bands are almost equally distributed, with an average sample interval of approximately 3nm, and each band’s full width at half maximum (FWHM) is roughly 7nm, as indicated by the manufacturer. During image acquisition, a three-dimensional data cube of size 512 $\times$ 512 $\times$ 204, with two spatial dimensions (X, Y) and one spectral dimension ($\lambda$), is formed^24^. Fig 1 depicts the idea of the hyperspectral cube. In the new version of the visualization, the axes of the hyperspectral cube have been labeled as Spatial dimension X (pixels) and Spatial dimension Y (pixels) for the two spatial axes, respectively, and Spectral dimension $\lambda$ (400 nm-1000 nm) for the spectral axis. Additionally, an indicative scale bar has been added to the cube, showing the spatial scale of 50 pixels. The spectral profile shows the reflectance curve of a pixel, with the spectral range labeled as “400 nm-1000 nm, 204 bands.” In a conceptual sense, the hyperspectral image can be viewed as comprising multiple two-dimensional slices, with each slice corresponding to the reflectance data at a given wavelength. Collectively, these slices can be said to form a data cube. At every pixel location in the image, the entire reflectance spectrum across all wavelengths is captured, thus enabling the precise identification of even slight variations in the chemical and optical properties of the stains.

**Fig. 2:**
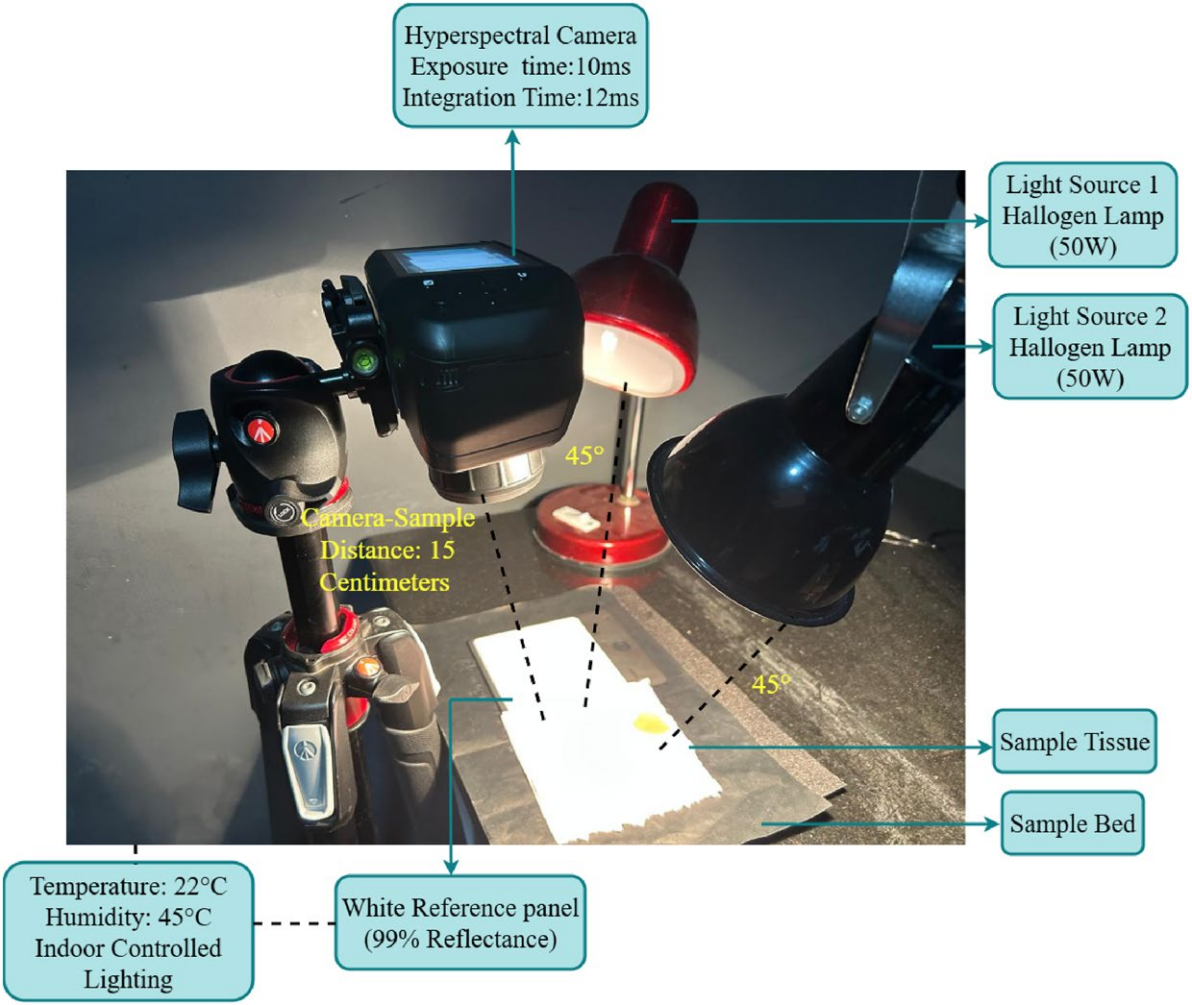
Experimental setup for beverage stain imaging with Specim IQ HSI camera.

The HSI system was set to ensure the consistency of the illumination and imaging conditions. Images were captured by the Specim IQ camera, which had a spectral range of 400–1000 nm and 204 spectral bands. The spatial resolution of the Specim IQ camera is 512 x 512 pixels. The Specim IQ camera is placed vertically 15 cm away from the sample. This distance is equivalent to a field of view of 12 x 12 cm. The hyperspectral imaging system is set by choosing the right settings for the exposure and integration of the image. In the Specim IQ hyperspectral imaging system, the exposure time of 10 ms determines the time for which the image sensor collects the incoming light. This affects the quality of the signal. On the other hand, the integration time of 12 ms is the total time required for the image acquisition cycle. This cycle includes the accumulation of the signal and the image sensor readout. This parameter is slightly higher than the exposure time to operate the image sensor. This parameter is set as recommended by the manufacturer. The sample was illuminated with two halogen lamps with a color temperature of approximately 3200 K, approximately 50 watts, and a broad spectrum illumination with the lamps positioned symmetrically at $\pm 45^\circ$ with respect to the sample surface and approximately 15 cm from the sample bed. Experiments were conducted in an air-conditioned atmosphere with a temperature set at $22 \pm 1~^\circ$C and the Relative Humidity (RH) was kept at $45 \pm 5\%$. Prior to image capture, the reflectance calibration was conducted with a Spectralon 99% diffuse white reference panel, and the dark current correction was applied. Reflectance calculation was done as follows:

1 $$\begin{aligned} R = \frac{I_{\textrm{raw}} - I_{\textrm{dark}}}{I_{\textrm{white}} - I_{\textrm{dark}}} \end{aligned}$$

where $I_{\textrm{raw}}$, $I_{\textrm{white}}$, and $I_{\textrm{dark}}$ represent raw signal, white reference, and dark reference, respectively (Fig. 2).

#### Representative spectral samples per class

As a further example of inter-class spectral variability, mean reflectance values were calculated at five representative wavelengths: 450, 550, 650, 750, and 900 nm. These values were chosen based on their high ANOVA F-scores. These wavelengths were chosen because they correspond to spectral regions that show high inter-class variance and therefore have high discriminative potential. Table 2 displays average values of reflection across every beverage class.

**Table 2:** Mean reflectance values at selected discriminative wavelengths for each beverage class.

Stain Class	450 nm	550 nm	650 nm	750 nm	900 nm	Dominant Discriminating Region
Papaya	0.72	0.81	0.84	0.89	0.93	Visible (450–600 nm)
Coffee	0.38	0.48	0.55	0.73	0.82	Blue–green (400–500 nm)
Pomegranate	0.21	0.19	0.35	0.62	0.71	Broad visible absorption
Orange	0.61	0.74	0.82	0.86	0.91	Visible edge (580–620 nm)
Tea	0.42	0.51	0.59	0.74	0.83	Blue–green (400–520 nm)
Wine	0.18	0.17	0.31	0.58	0.68	Broad visible absorption
Whisky	0.65	0.71	0.75	0.82	0.88	Near-infrared (750–900 nm)
Rum	0.63	0.69	0.73	0.80	0.85	Near-infrared (750–900 nm)
Brandy	0.68	0.75	0.79	0.87	0.92	Visible–NIR transition

The largest inter-class differences occur in the visible spectrum (400–700 nm), where chromophores associated with pigmentation have unique absorption characteristics. Pomegranate and wine have consistently low reflectance values ($<0.35$) in the visible spectrum, consistent with their anthocyanin-dominated spectral profiles. By contrast, papaya and orange have high reflectance values ($>0.60$) in the 450–650 nm spectral region.

Coffee and tea have similar reflectance within the visual range, but they are mostly distinct in the range of 450-530nm, where different patterns of absorption can be seen. Finally, rum and whisky are the drinks that are most comparable in terms of their spectra. The most significant difference between them occurs in the near-infrared band (750 nm-900 nm). That is also shown by all of the confusion matrices for all the models, which show that the coffee and tea and rum whisky pairs have the highest rates of misclassification. The deep learning algorithms can tell the drinks apart because they can see the distinct patterns in reflectance at different wavelengths.

### Sample preparation and experimental protocol

Wine, Whisky, Rum, Brandy, Papaya juice, Coffee, Pomegranate juice, Orange juice, and Tea have been proposed as options for the beverage classes. The selection of nine classes of beverages was carried out in a manner that provided a balanced and controlled experimental design while covering a wide range of spectral characteristics of interest in a forensic context. The selected classes include those that are similar in appearance and those that are difficult to classify due to similar spectral characteristics, i.e., coffee-tea and rum-whisky, as they tend to show similar spectral behavior. While there have been studies on using a larger number of stain classes in the literature, in this work, there was an emphasis on controlled variation in terms of using multiple substrates (white, pink, brown) and geometric configurations (flat and folded) as well as time evolution (0–5 hours). This approach provides a more realistic evaluation of classification accuracy rather than relying on increasing the number of classes. Each stain was applied in the form of a droplet of volume 1 mL with the aid of a micro-pipette that has an accuracy of $\pm 0.05$ mL. The stains were applied on white, pink, and brown substrates. The volume of each droplet was kept constant for all substrates to ensure that the stain morphology is similar for all. For each class of beverages, $n = 3$ independent samples were considered.

All experiments were performed inside and under controlled lighting and temperature conditions. To observe the effects of geometry on the spectral behavior of the substrates, substrates in flat and folded forms were considered.

**Fig. 3:**
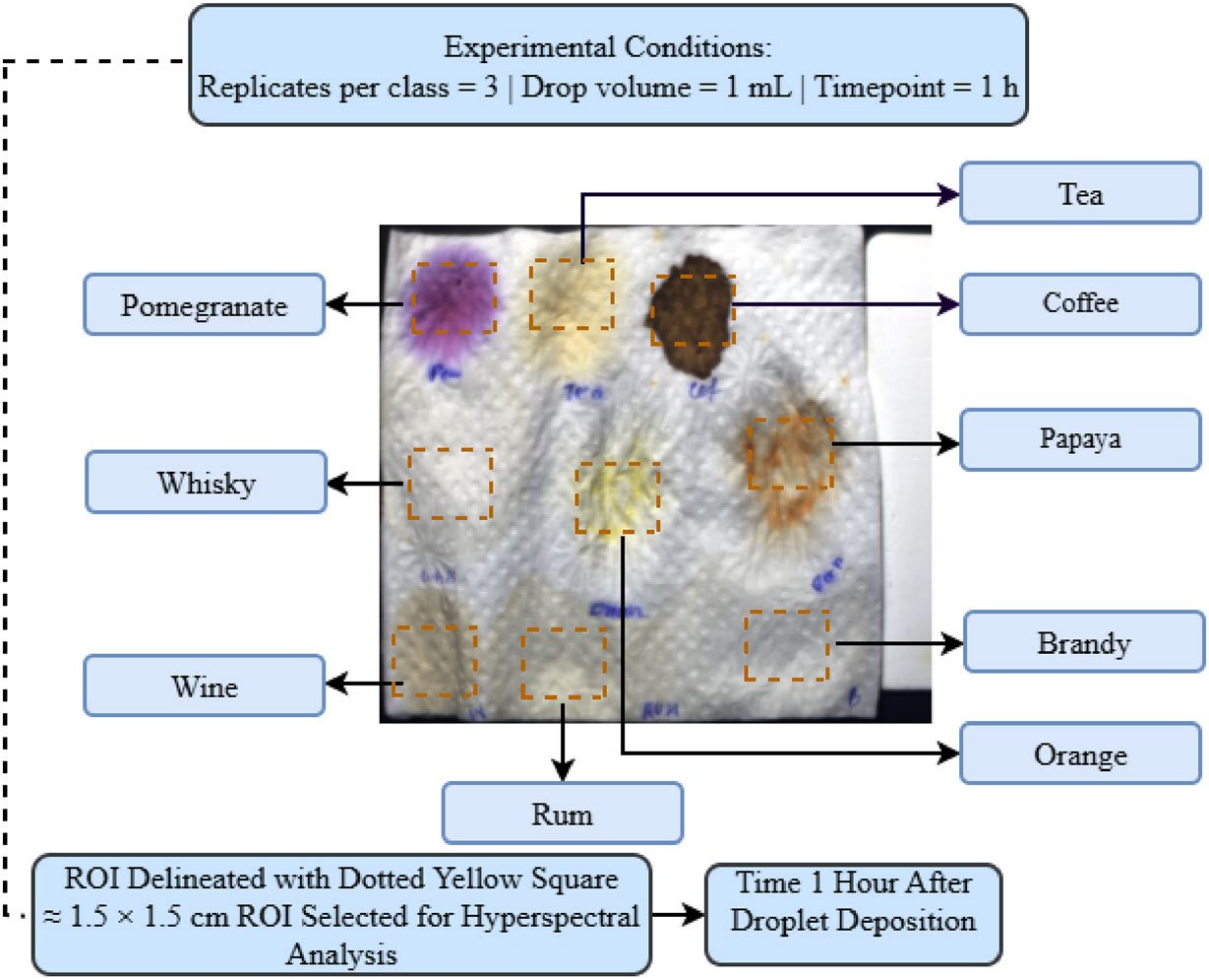
Labeled beverage stains on white tissue for HSI acquisition.

Temporal evolution is an important aspect for characterizing the stain because of absorption, evaporation, and oxidation effects. Hyperspectral images are acquired at six different time intervals to observe temporal evolution. The first acquisition was performed immediately after applying the stain on the substrates ($t = 0$ minutes), where *t* represents the time elapsed after applying the stain. The subsequent acquisitions are performed after every 1 hour up to 5 hours ($t = 1$ to 5 hours). Out of these, the 1-hour time point has been chosen as the primary reference for analysis and further comparative analysis. The rationale for choosing the 1-hour time point is based on the fact that the spectral signatures tend to behave in a stable manner after the initial phase of absorption and partial evaporation, thus reducing the variability compared to the immediate time point, i.e., $t=0$. In Fig. 3, the labeled stains of the beverage on the white tissue paper are depicted, where the time point for the image is taken as $t=1$ hour after the application of the stains. The Regions of Interest (ROI) were identified using the Specim IQ software, where rectangular regions were selected for each stain, ensuring the exclusion of specular reflections and the boundary areas. The selected ROI is depicted as dashed yellow squares in the figure, with an area of $1.5 \times 1.5$ cm. The stains were monitored over time to observe the natural spectral evolution across different substrates. This process is also useful for validating previously established trends for hyperspectral analysis of aging stains^25^. As described in^26^, consideration of variations in substrates improves the robustness of the dataset for heterogeneous conditions in real-world scenarios. The variety of stains and substrates within this dataset aids in the development of classification methods for realistic forensic applications.

### Feature extraction and formatting

Spectral data for every pixel across the 204 hyperspectral bands were extracted and stored as Comma Separated Values (CSV) files to make the numerical analysis easier later on. Overall, total 54 CSV files were generated, categorized by tissue type (white, pink, or brown) and fold condition (flat or folded). The data underwent preprocessing steps that included removing noise and standardizing and correcting potential inconsistencies. All files were then merged to create a single dataset of 121,496 spectral samples. Each sample has 204 spectral attributes and is tagged with a class label for one of the nine beverage stains. Labeling is as follows: 0 for Papaya, 1 for Coffee, 2 for Pomegranate, 3 for Orange, 4 for Tea, 5 for Wine, 6 for Whisky, 7 for Rum, and 8 for Brandy. This data, with all of its details and structure, was the base dataset used to train supervised deep learning models and evaluating their classification performance within the beverage stain identification domain, specifically to test their ability to identify various beverage stains using well-established hyperspectral analysis methods.

#### Bias assessment and mitigation

To assess the possibility of any bias present in the dataset, a series of validation strategies were employed. First, the proportion of each class was checked among the nine beverage categories. It was found that the proportion was almost balanced with a variation of less than 3%. The samples were collected on multiple substrate types and configurations, which ensured the diversity of the samples. Additionally, the temporal variation was uniformly carried out on six intervals (0–5 hours), which ensured the avoidance of any particular stage of aging. Stratified sampling was employed during hold-out and cross-validation strategies. Additionally, the ANOVA score was recalculated for each fold during the cross-validation approach on the training data only. The macro-averaging approach was employed for the evaluation metrics, which ensured the consideration of all the classes equally. All the above strategies ensured the avoidance of class imbalance, substrate bias, temporal bias, and evaluation bias.

### Feature selection using ANOVA test and band optimization analysis

To reduce spectral redundancy while preserving discriminative information, an ANOVA test was employed to figure out the most meaningful spectral bands from the original 204-band hyperspectral dataset. This work used ANOVA-based band selection as a supervised feature selection method, utilizing class labels for various beverage stains to assess the discriminative capability of each spectral band. It evaluates the discriminative capability of each spectral band by comparing the inter-class variance with the intra-class variance.

For a spectral feature $x_j$, the F-statistic is defined as:

2 $$\begin{aligned} F_j = \frac{MSB_j}{MSW_j} \end{aligned}$$

where $MSB_j$ (mean square between groups) and $MSW_j$ (mean square within groups) are computed as:

3 $$\begin{aligned} & MSB_j = \frac{\sum _{k=1}^{K} n_k (\bar{x}_{k,j} - \bar{x}_j)^2}{K - 1} \end{aligned}$$

4 $$\begin{aligned} & MSW_j = \frac{\sum _{k=1}^{K} \sum _{i=1}^{n_k} (x_{i,k,j} - \bar{x}_{k,j})^2}{N - K} \end{aligned}$$

Here:

- *K* shows the total quantity of beverage classes,
- $n_k$ represents the total amount of samples in class *k*,
- $\bar{x}_{k,j}$ is the mean value of feature *j* within class *k*,
- $\bar{x}_j$ is the global mean of feature *j* across all samples,
- *N* represents the total number of samples.

A higher value of $F_j$ indicates stronger class separability for the corresponding spectral band.

#### Band ranking and optimal feature count determination

After computing the F-statistics for all 204 spectral bands (performed strictly within training folds to prevent information leakage), the bands were ranked in descending order according to their discriminative strength.

A 5-fold cross-validation research was performed utilizing the MLP classifier to ascertain the best number of selected bands. Classification accuracy was evaluated for progressively increasing subsets of top-ranked spectral bands.

**Fig. 4:**
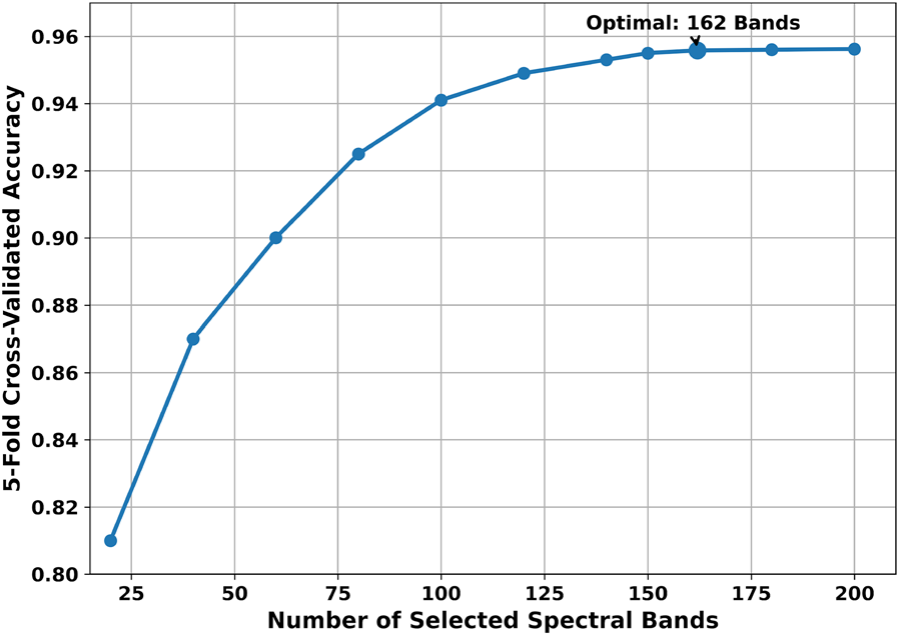
Accuracy versus number of selected spectral bands based on ANOVA ranking using 5-fold cross-validation.

As shown in Fig 4, classification accuracy increases rapidly When the quantity of chosen bands grows from 20 to 100, indicating that the highest-ranked bands capture the majority of discriminative spectral information. The improvement becomes gradual between 100 and 150 bands, and performance stabilizes beyond approximately 162 bands, demonstrating diminishing returns.

The best stable performance was obtained with 162 selected spectral bands, which produced a 5-fold cross-validation accuracy of 95.58%.

Beyond 162 bands, the gain in accuracy was marginal (less than 0.1%), while computational complexity increased. Therefore, 162 bands were chosen as the optimal compromise among classification efficacy and dimensionality reduction.

#### Final feature matrix

From the initial extraction, a total of 121,496 spectral samples were obtained for all the defined regions of interest. During the preprocessing stage, a total of 4,157 spectral samples were discarded, representing 3.4% of the total, based on the following criteria: (i) spectral bands where more than 5% of the values were missing or saturated pixel values, resulting in the removal of 2,891 spectral samples, and (ii) duplicate pixel values resulting from the overlapping regions of interest for the folded tissue conditions, resulting in the removal of 1,266 spectral samples. These removals did not preferentially target any specific class of beverages, substrate types, or time points, where the variation was less than 3%. After the quality filtering and feature selection using the ANOVA test, the final spectral data set consisted of 117,339 spectral samples, each described by 162 spectral features. The spectral data set’s feature matrix can be defined by the following equation:

5 $$\begin{aligned} \textbf{X} \in \mathbb {R}^{117{,}339 \times 162} \end{aligned}$$

This decreased feature space not only ensures the convergence of deep learning.

### Spectral signature analysis of beverage stains

The spectral signature of the nine beverage stains was analyzed across the wavelength spectrum, extending from 400 nm to 1000 nm. The hyperspectral data were captured at six time intervals, namely 0 min, 1 hr, 2 hr, 3 hr, 4 hr, and 5 hr, to analyze the spectral characteristics over time, i.e., the drying process. To illustrate the spectral characteristics for better understanding and comparison, the spectral analysis is provided for the 1-hour time interval, at which the reflectance values were steady after the initial absorption and partial evaporation process.

**Fig. 5:**
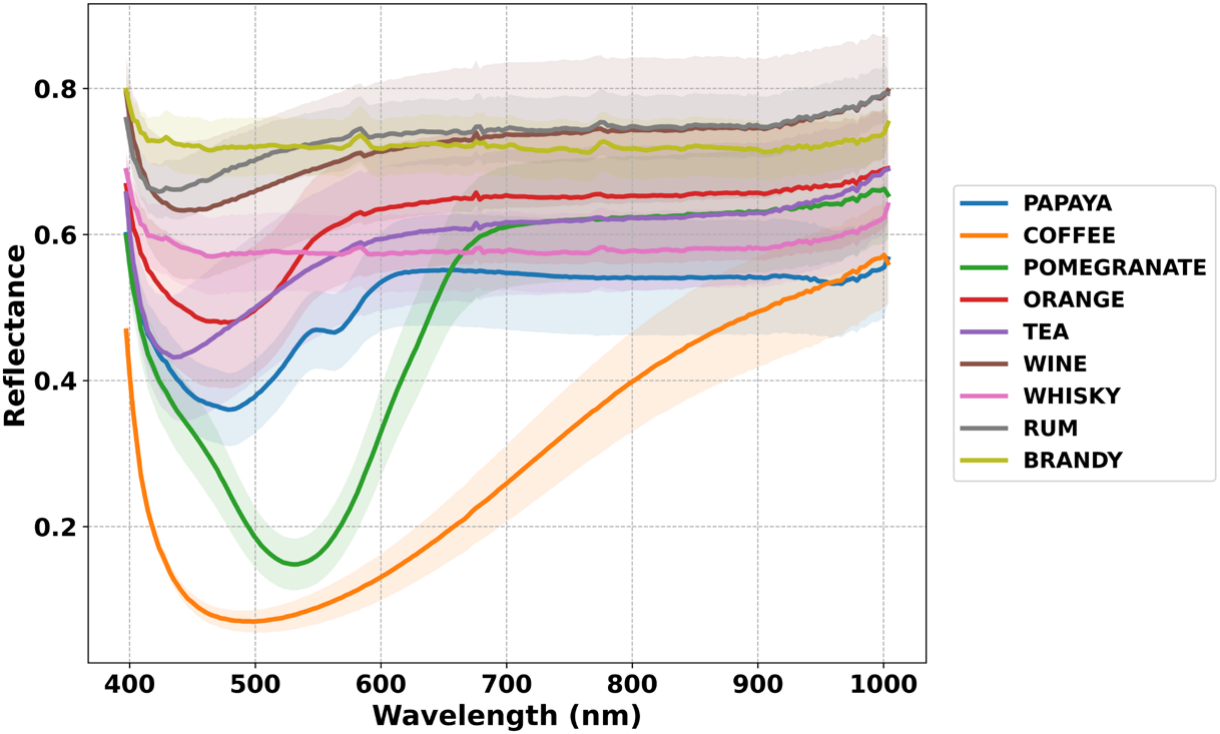
Mean spectral signatures of beverage stains on white tissue at 1 hour (random ROI).

**Fig. 6:**
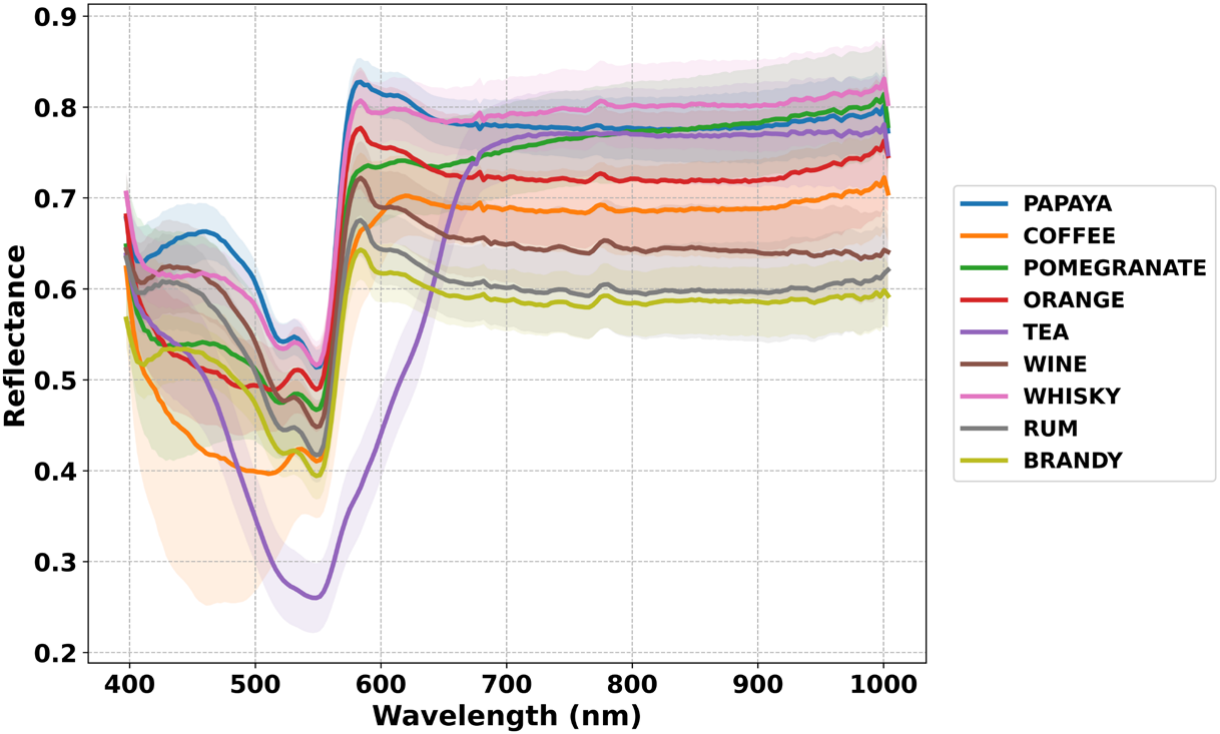
Mean spectral signatures of beverage stains on pink tissue at 1 hour (random ROI).

**Fig. 7:**
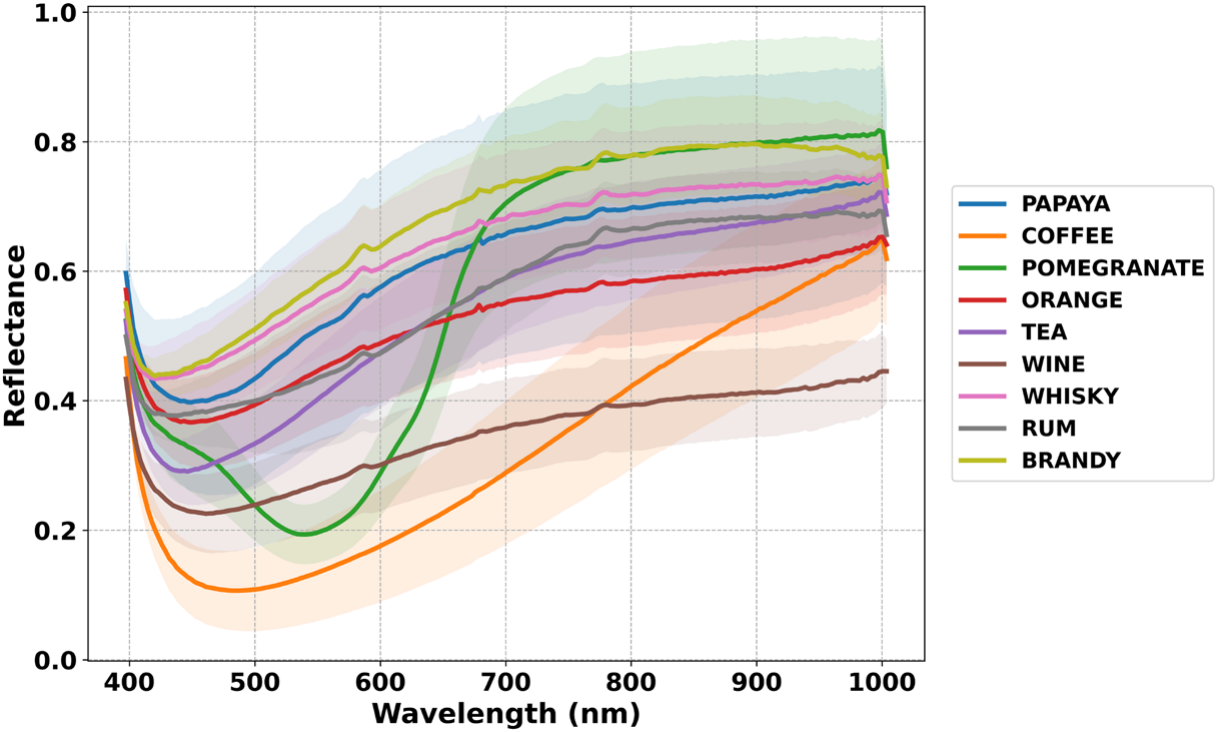
Mean spectral signatures of beverage stains on brown tissue at 1 hour (random ROI).

Figure 5 displays the spectral signature obtained from the ROI for the white tissue sample. To validate the robustness of the results, the ROI was randomly chosen for the pink and brown tissues, as depicted in Fig. 6 and Fig. 7, respectively. The spectral trends obtained for the randomly chosen ROI indicate that the reflectance characteristics are not related to the pixel location but to the beverage composition itself. In general, the most significant variation in reflectance values in all tissues was found in the visible region, ranging from 400 nm to 700 nm. This variation is due to pigment or chromophore absorption. Pomegranate and wine have low reflectance values in the blue-green region. This may be due to the presence of anthocyanins. For coffee and tea exhibit moderate absorption due to melanoidins and polyphenolic content. A substantial variation in reflectance values occurs in the red edge region, ranging from 580 nm to 620 nm, found in most of the beverages. In the near-infrared region, ranging from 700 nm to 1000 nm, an increase in reflectance values was found. Lighter-colored drinks, such as orange and brandy, show relatively high reflectance values, while darker-colored stains show relatively flat reflectance values. Rum and whisky show similar spectral characteristics throughout the visible light and near-infrared spectra. This may be the reason why there was minor classification overlap in the subsequent confusion matrix analysis. Thus, the repeatability of spectral patterns on multiple tissues and random ROI selection confirms that hyperspectral imaging accurately captures the intrinsic chemical and optical differences in the beverage stains, making it a reliable tool in non-destructively classifying the samples.

### Overview

**Fig. 8:**
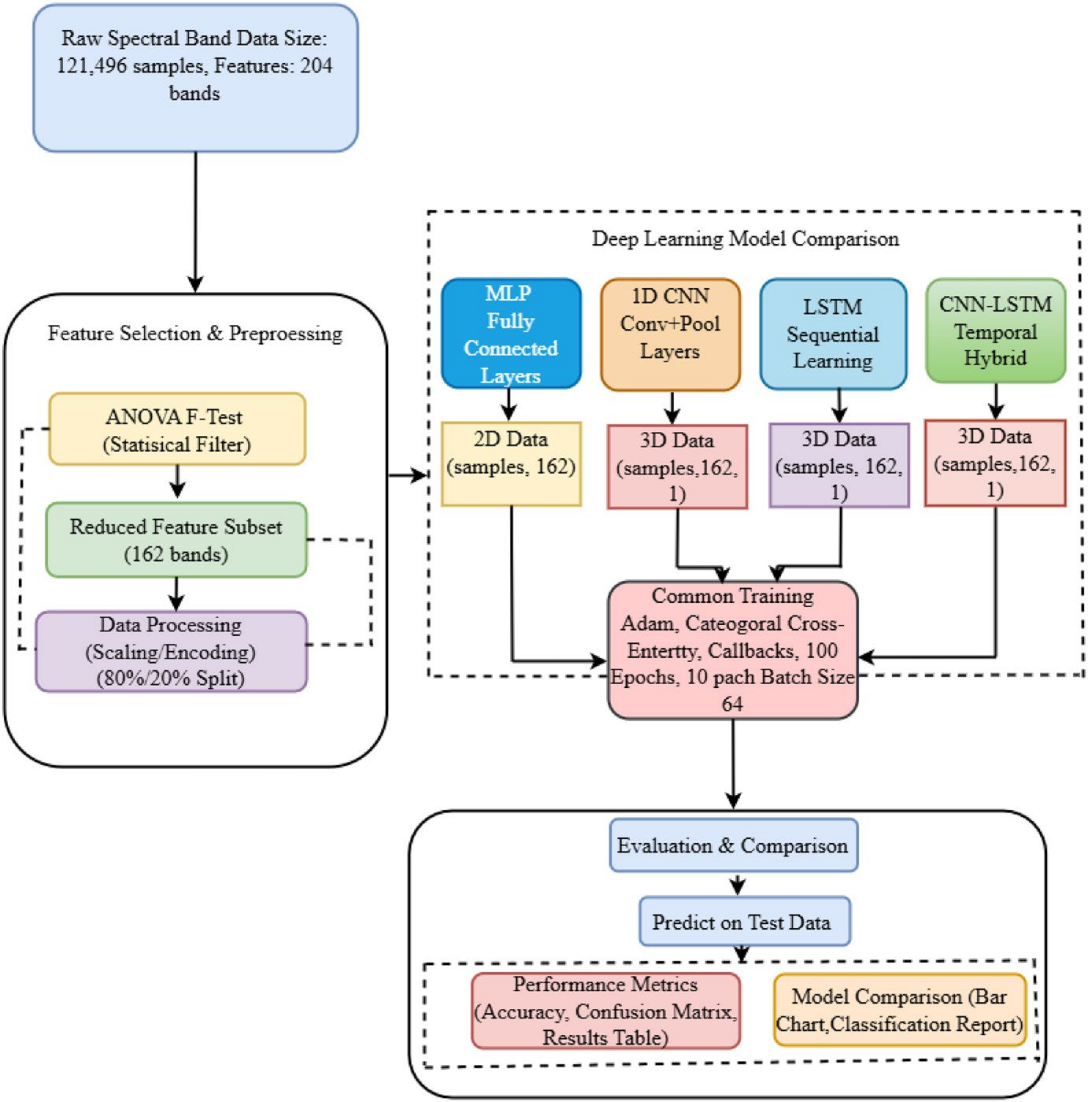
Workflow of the proposed hyperspectral deep learning framework for stain classification.

The selected 162 spectral bands obtained from the ANOVA test were used as input features to train four deep learning architectures MLP, 1D-CNN, LSTM, and a hybrid CNN–LSTM model. The hybrid architecture combined local feature extraction with sequential pattern learning, thereby enhancing the overall understanding of spectral characteristics associated with different stains. Figure 8 illustrates the proposed deep learning framework for beverage stain classification using hyperspectral imaging and ANOVA-based feature selection.

#### Train–test split strategy

The final cleaned dataset, including 117,339 spectral samples, was divided into a training set and test set using an 80:20 stratified split ratio based on pixels. This gave us around 93,865 training samples and 23,474 independent test samples. In training the model, 10% of the training dataset was set aside for validation through setting the parameter “validation_split” to 0.1. Consequently, the effective training distribution corresponded to approximately 72% training, 8% validation, and 20% testing relative to the full dataset. Final performance metrics and statistical significance testing were computed entirely on the reserved test set. For this study, the classification problem is posed as a pixel-wise spectral classification problem, in which each pixel is considered as an individual spectral sample. This is consistent with conventional approaches in analyzing hyperspectral images and is intended to test the discriminative power of spectral signatures. However, it has also been recognized that pixel-wise random splitting may create some similarity between the training and test samples with respect to the same stain instance, which may result in biased performance evaluation. Although the data set has variability with respect to diverse substrate types, configurations, and time intervals (0–5 hours), thus ensuring considerable spectral diversity, the evaluation with group splitting based on stain instances will also be considered for the future direction of work.

### Proposed model

The proposed framework has been developed to achieve efficient and accurate classification of beverage stains using statistical feature selection and deep learning techniques. After preprocessing the hyperspectral images, an ANOVA-based band selection method was employed to identify the most discriminative features. Using this method, 162 informative spectral bands were obtained, which reduced the dimensionality of the data while retaining the spectral features necessary to differentiate between visually similar beverage stains. Utilizing the optimized features, four different architectures of deep learning models were developed and compared, including MLP, 1D-CNN, LSTM, and a hybrid CNN-LSTM model. Every model was run in similar experimental settings and contrasted with one another. This study utilized the Adam optimizer with an initial learning rate of 0.001. Early stopping approaches and a learning rate decrease were used to prevent overfitting. The categorical cross-entropy loss function was used to train the models. The problem was approached as a pixel-wise spectral classification problem since the hyperspectral image is a 3D-cube, along with two dimensions providing the spatial information and one representing the spectral information. In this problem, the spectral vectors obtained from the well-defined regions of interest were treated as one-dimensional reflectance sequences. Although the 3D-CNN model is effective in the joint consideration of spatial and spectral information, the consideration of spatial information in this problem could introduce substrate texture, background patterns, and illumination variation factors, which are not related to the intrinsic spectral characteristics of the beverage stains. In addition, the 3D-CNN model is computationally complex. The excellent results obtained by the MLP and 1D-CNN models indicate that the discriminative features in the dataset are in the spectral domain. The hyperparameters of the models used in the experiment are given in Table 3. The sum of all count of trainable parameters for each model is also documented to elucidate the complexity and computational expense of the model.

**Table 3:** Hyperparameter configuration of deep learning models used for beverage stain classification.

Parameter	MLP	1D-CNN	LSTM	CNN- LSTM
Optimizer	Adam ($\beta _1$=0.9, $\beta _2$=0.999)	Adam	Adam	Adam
Initial learning rate	0.001	0.001	0.001	0.001
LR scheduler	ReduceLROnPlateau (factor=0.5, patience=5)	Same	Same	Same
Batch size	64	64	64	64
Max epochs	100	100	100	100
Early stopping patience	10 epochs (monitor: val_loss)	Same	Same	Same
Loss function	Categorical cross-entropy	Same	Same	Same
Dropout rate	0.3 (after each dense layer)	0.3	0.3	0.3
Hidden layers (MLP)	512 $\rightarrow$ 256 $\rightarrow$ 128 $\rightarrow$ 9 (softmax)	—	—	—
Conv filters (CNN)	—	64(k=3), 128(k=3)	—	64(k=3)
LSTM units	—	—	128	64
Input normalization	StandardScaler (zero mean, unit variance)	Same	Same	Same
Noise removal	Bands with >5% missing values removed; median imputation for residual NaNs	Same	Same	Same
Activation (hidden)	ReLU	ReLU	tanh/ sigmoid gates	ReLU +tanh
Framework	TensorFlow/Keras 2.12	Same	Same	Same
**Total trainable** **parameters**	$\approx$ ** 230,000**	$\approx$ ** 120,000**	$\approx$ ** 150,000**	$\approx$ ** 95,000**

#### Mathematical formulation and design of deep learning models

After running the ANOVA F-test, we picked out the 162 most useful spectral bands, which shrank the feature matrix down to $X \in \mathbb {R}^{N \times 162}$–*N* being the number of samples. Then we trained four different deep learning models on this trimmed-down data: a plain MLP, a 1D-CNN, an LSTM, and a combo CNN–LSTM. Each model learns how to map *X* to *Y*, where $Y = \{0,1,2,\dots ,8\}$ stands for the nine different beverage stain classes^27–29^.

#### Multi-layer perceptron (MLP)

An MLP is a type of neural network that sends information forward. Here’s the basic idea: each neuron takes its inputs, combines them in a linear way, and then passes the result through a nonlinear activation function. So, for each layer, we will get something like this:

6 $$\begin{aligned} z_i = W_i x + b_i, \quad a_i = f(z_i) \end{aligned}$$

Here’s what all those symbols mean:

- *x* signifies the input feature vector,
- $W_i$ and $b_i$ represent the weight matrix and bias term of the $i^{th}$ layer,
- $f(\cdot )$ shows the activation function, specifically implemented as ReLU, defined by
- $$f(x) = \max (0, x)$$, and
- $a_i$ represents the output following to the activation process.

At the end, the network produces some numbers, and We apply the SoftMax function to convert these into probabilities for each class:

7 $$\begin{aligned} P(y=k|x) = e^{z_k} {\sum _{j=1}^{C} e^{z_j}} \end{aligned}$$

C is 9, which is the same as the number of sorts of stains we have to deal with. We change the weights and biases of the network so that the predictions are correct. We do this by making the categorical cross-entropy loss as small as possible:

8 $$\begin{aligned} L = -\frac{1}{N} \sum _{i=1}^{N} \sum _{k=1}^{C} y_{i,k} \log (\hat{y}_{i,k}) \end{aligned}$$

In this case, $y_{i,k}$ is the real class label and $\hat{y}_{i,k}$ is what the model thinks it is. The goal is to make those predictions as near to the truth as feasible.

#### 1D-convolutional neural network (1D-CNN)

A 1D-CNN automatically takes up local spectral features by using filters that move along the spectral dimension. 2D and 3D-CNNs normally work with geographic data, however 1D-CNNs only with spectral data. That’s why it works so well for hyperspectral vectors that are only one -dimensional.

This is how every convolutional layer works:

9 $$\begin{aligned} h_j^{(l)} = f\left( \sum _{i=1}^{m} w_i^{(l)} x_{j+i-1} + b^{(l)}\right) \end{aligned}$$

In this case, *m* is the size of the kernel, $w_i^{(l)}$ is the filter weights, and $b^{(l)}$ is the bias. Then, the activations $h_j^{(l)}$ go through a ReLU non-linearity, which essentially means that the network becomes better at finding complicated patterns. The model employs max pooling to maintain the strongest spectrum responses and make the data smaller:

10 $$\begin{aligned} p_j = \max (h_{2j-1}, h_{2j}) \end{aligned}$$

After that, these spectral properties are turned into a one -dimensional vector and sent through fully connected layers to be classified. This arrangement helps the model focus on local features and truly get the essential spectral patterns right. That’s a key part of why it does so well on the hyperspectral dataset.

#### Long short term memory (LSTM)

LSTM networks are made to find long-term relationship in sequences. On the other hand, HSI comprises neighboring spectral bands that are very similar to each other. In these cases, LSTM will understand how these bands are related: they will follow the smooth changes and show how bands are related over vast distances. The decision of what data to retain and what to discard is made via a set of gates located within each LSTM cell:

11 $$\begin{aligned} f_t&= \sigma (W_f [h_{t-1}, x_t] + b_f) \end{aligned}$$

12 $$\begin{aligned} i_t&= \sigma (W_i [h_{t-1}, x_t] + b_i) \end{aligned}$$

13 $$\begin{aligned} \tilde{C}_t&= \tanh (W_c [h_{t-1}, x_t] + b_c) \end{aligned}$$

14 $$\begin{aligned} C_t&= f_t \odot C_{t-1} + i_t \odot \tilde{C}_t \end{aligned}$$

15 $$\begin{aligned} o_t&= \sigma (W_o [h_{t-1}, x_t] + b_o) \end{aligned}$$

16 $$\begin{aligned} h_t&= o_t \odot \tanh (C_t) \end{aligned}$$

The forget, input, and output gates are represented by $f_t$, $i_t$, and $o_t$. $C_t$ is the memory of the cell, and $\odot$ is simple multiplication of elements. These gates enable the LSTM choose which spectral information to keep and which to throw away as it goes through the data. In this manner, it learns the important patterns across wavelength and actually understands how distinct spectral bands are related to each other.

#### CNN–lstm hybrid model

The CNN-LSTM hybrid takes the best parts of both architectures: CNN’s capacity to find localized spectral features and LSTM’s ability to describe sequential relationships. This integration looks for spectral patterns that are both short-range and long-range, which should make it an excellent choice for classifying hyperspectral stains. But the extra complexity in the architecture didn’t actually help with this dataset. Maybe it overfit, or maybe there just aren’t strong temporal links between the spectral bands here. Initially, 1D convolutional layers extract local spectral features:

17 $$\begin{aligned} h_t = \textrm{Conv1D}(x_t) \end{aligned}$$

These features are then passed into an LSTM layer to model their sequential dependencies:

18 $$\begin{aligned} H = \textrm{LSTM}(h_t) \end{aligned}$$

Finally, the classification layer applies the Softmax function:

19 $$\begin{aligned} \hat{y} = \textrm{Softmax}(W_H H + b_H) \end{aligned}$$

Although this hybrid architecture effectively combines convolutional encoding and recurrent sequence learning, its performance in this study was comparatively lower than the individual MLP, 1D-CNN, and LSTM models. So even though CNN–LSTM sounds great in theory for spectral–spatial learning, simpler models like MLP and 1D-CNN actually worked better and gave more stable results for this particular hyperspectral forensic task.

### Evaluation metrics and results

The proposed deep learning architectures were evaluated for beverage stain classification using four standard performance metrics: Accuracy, Precision, Recall, and F1-score. These metrics were computed from the confusion matrix for each model across the nine beverage classes.

The mathematical formulations of the evaluation metrics are defined as follows:

20 $$\begin{aligned} \text {Accuracy}&= \frac{TP + TN}{TP + TN + FP + FN} \end{aligned}$$

21 $$\begin{aligned} \text {Precision}_c&= \frac{TP_c}{TP_c + FP_c}, \quad \text {Recall}_c = \frac{TP_c}{TP_c + FN_c} \end{aligned}$$

22 $$\begin{aligned} \text {F1}_c&= 2 \times \frac{\text {Precision}_c \times \text {Recall}_c}{\text {Precision}_c + \text {Recall}_c} \end{aligned}$$

TP, TN, FP, and FN shows the total counts of true positives, true negatives, false positives, and false negatives across all classes. These values are utilized to compute the accuracy. TP_c, FP_c, FN_c shows the true positives, false positives, and false negatives for class C, respectively.

Macro-averaged metrics were reported to ensure balanced evaluation across all nine beverage categories, assigning equal importance to each class irrespective of minor variations in sample distribution.

#### Overall model performance

Table 4 summarizes the overall macro-averaged performance of the four deep learning architectures.

**Table 4:** Comparison of deep learning models for beverage stain classification.

Model	Accuracy	Precision (Macro)	Recall (Macro)	F1-Score (Macro)
MLP	0.9558	0.9554	0.9555	0.9553
1D-CNN	0.9524	0.9519	0.9518	0.9517
LSTM	0.9451	0.9497	0.9497	0.9496
CNN-LSTM	0.9033	0.9049	0.9047	0.9046

All models achieved strong classification performance. The MLP demonstrated the highest overall accuracy (95.58%), followed closely by 1D-CNN and LSTM. The CNN-LSTM hybrid model exhibited comparatively lower performance, likely due to increased architectural complexity and optimization challenges.

#### Per-class performance analysis

A comprehensive class-wise evaluation was conducted, calculating precision, recall, and F1-scores for each class in the optimal model (MLP), as shown in Table 5.

**Table 5:** Measures of MLP model performance per class.

Class	Support	Precision	Recall	F1-score
Papaya	2621	0.9814	0.9885	0.9849
Coffee	2637	0.9231	0.9392	0.9311
Pomegranate	2700	0.9780	0.9669	0.9724
Orange	2625	0.9739	0.9847	0.9793
Tea	2550	0.9724	0.9667	0.9695
Wine	2550	0.9644	0.9769	0.9706
Whisky	2539	0.9567	0.9661	0.9614
Rum	2625	0.9527	0.9531	0.9529
Brandy	2627	0.9189	0.9237	0.9213
**Macro Avg.**	–	0.9554	0.9555	0.9553

While most beverage classes achieved F1-scores above 0.95, Coffee (0.9311) and Brandy (0.9213) exhibited comparatively lower performance. This observation is consistent with spectral similarity between Coffee–Tea and Brandy–Rum pairs, which increases inter-class confusion.

#### 5-Fold stratified cross-validation

In order to further test the robustness of the model, 5-fold stratified cross-validation was applied on the training set from the stratified 80:20 split. Within each fold, ANOVA-based band selection was recomputed exclusively on the corresponding training partition to prevent information leakage. The fold-wise accuracies and summary statistics are presented in Table 6.

**Table 6:** 5-fold stratified cross-validation accuracies for deep learning models.

Model	Fold 1	Fold 2	Fold 3	Fold 4	Fold 5	Mean ± Std
MLP	0.9541	0.9563	0.9571	0.9548	0.9567	0.9558 ± 0.0012
1D-CNN	0.9508	0.9531	0.9519	0.9522	0.9540	0.9524 ± 0.0012
LSTM	0.9439	0.9461	0.9455	0.9447	0.9453	0.9451 ± 0.0008
CNN–LSTM	0.9012	0.9041	0.9038	0.9027	0.9048	0.9033 ± 0.0014

The consistently low standard deviations across folds ($\le 0.0014$ for all models) indicate strong stability and robustness with respect to sampling variation. The MLP achieved the highest mean cross-validated accuracy (95.58%), followed by 1D-CNN and LSTM.

Importantly, the cross-validation results closely align with the independent held-out test performance reported earlier, thereby confirming the reliability, generalization capability, and statistical consistency of the proposed hyperspectral classification framework.

#### Statistical significance testing

To determine the statistical significance of the reported performance disparity between models, two complementing statistical tests were performed.

***Paired t-test on 5-Fold cross-validation***

A two-tailed paired t-test ($\alpha = 0.05$, $n = 5$ folds) was applied to fold-wise cross-validation accuracies. The test statistic is defined as:

23 $$\begin{aligned} t = \frac{\bar{d}}{s_d / \sqrt{n}}, \quad d_k = Acc_A^{(k)} - Acc_B^{(k)} \end{aligned}$$

where $d_k$ denotes the difference in classification accuracy between two models on fold *k*, $Acc_A^{(k)}$ and $Acc_B^{(k)}$ represent the accuracies of models *A* and *B* on the *k*-th fold, $\bar{d}$ denotes the mean accuracy difference across all folds, and $s_d$ represents the standard deviation of the fold-wise differences.

The t-statistic in the Table 7 is the value that was computed from the paired t-test to find the difference between the mean cross-validation accuracy of two models. The p-value shows how likely it is that the difference happened by accident when the null hypothesis says that the models work the same way. The difference in performance of the models is statistically important because the p-value is lower than the significance criterion ($\alpha = 0.05$). Finally, the degrees of freedom (df) are found by taking $n-1$, where *n* is the number of folds in the cross-validation ($n=5$), which gives $df=4$. Cohen’s *d* is the standardized effect size, which shows how big the difference in performance is between two models in terms of standard deviation units.

**Table 7:** Paired t-test results across 5-fold cross-validation accuracies.

Comparison	t-statistic	p-value	df	Cohen’s d
MLP vs 1D-CNN	3.052	0.0379	4	2.534
MLP vs LSTM	13.607	0.0002	4	9.385
MLP vs CNN-LSTM	70.710	$<0.0001$	4	36.0
1D-CNN vs LSTM	13.424	0.0002	4	6.403
1D-CNN vs CNN-LSTM	53.241	$<0.0001$	4	33.7
LSTM vs CNN-LSTM	91.313	$<0.0001$	4	32.8

All pairwise comparisons were statistically significant ($p < 0.05$), indicating that performance differences are not attributable to random fold variation.

***McNemar test on test set predictions***

To further validate model superiority on the held-out test set ($N_{test} = 23{,}474$), McNemar’s test with continuity correction was applied:

24 $$\begin{aligned} \chi ^2 = \frac{(|b - c| - 1)^2}{b + c} \end{aligned}$$

In the ones mentioned above, equation, *b* denotes the quantity of samples accurately classified by the MLP but misclassified the alternative model, and *c* signifies the quantity of samples correctly classified by the alternative model but misclassified by the MLP. The McNemar chi-square statistic $\chi ^2$ assesses if the disparity in error rates between the two models is statistically significant. The McNemar test outcomes for the pairwise model comparison are displayed in Table 8.

**Table 8:** McNemar test results on test-set predictions.

Comparison	b	c	$\chi ^2$	Significance
MLP vs 1D-CNN	179	100	21.81	$p<0.001$
MLP vs LSTM	364	113	131.03	$p<0.001$
MLP vs CNN-LSTM	1332	100	1058.21	$p<0.001$

The *p*-value refers to the significance level, which indicates how likely it is that the difference between the predictions of the models was just by chance. The exceedingly low *p*-value of $p < 0.001$ signifies a statistically significant disparity in the performance of the models.

#### Model-wise analysis

The confusion matrices for each architecture give a lot of information on how well the model can classify things between classes. The diagonal elements show right predictions, and the off-diagonal elements show wrong predictions.

**Fig. 9:**
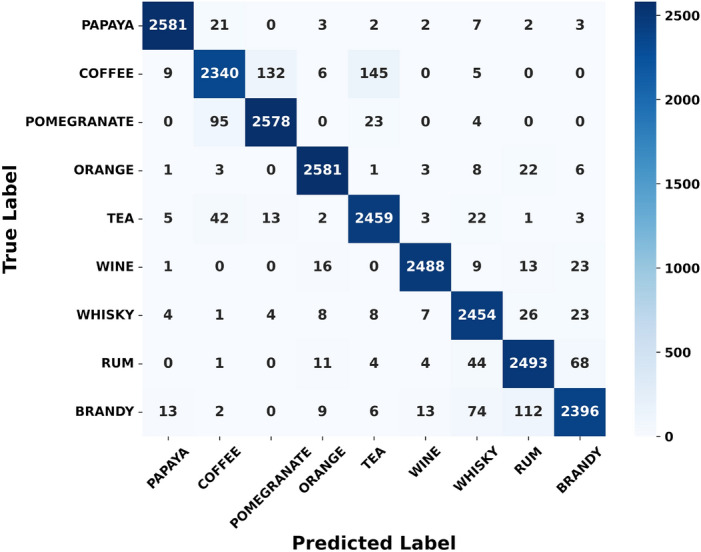
Confusion matrix of the MLP model for beverage stain classification.

**Fig. 10:**
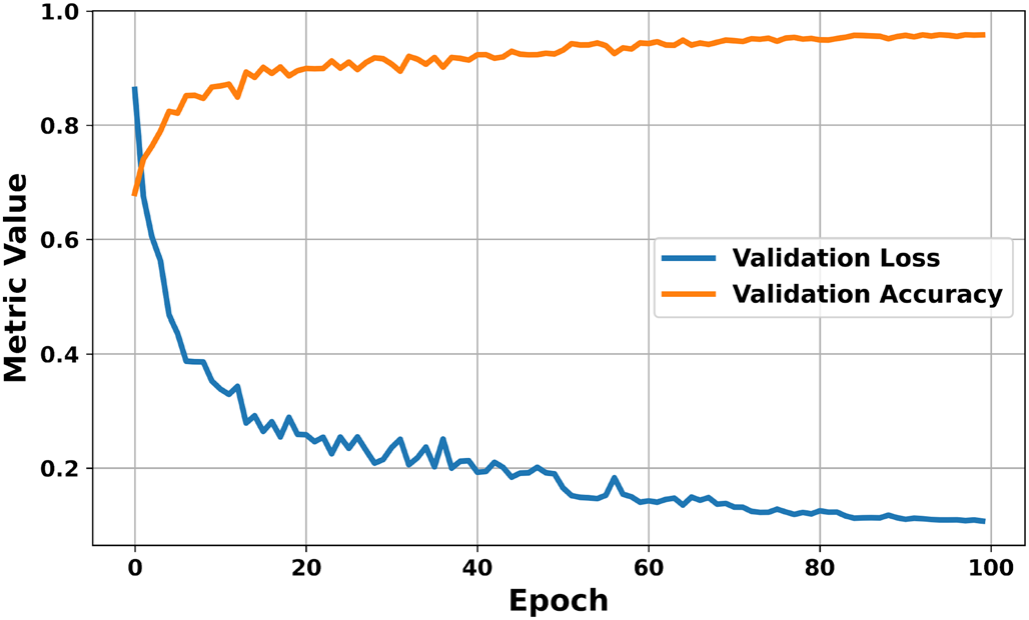
Validation loss and validation accuracy curves for the MLP model.

***MLP model*** The diagonal dominance of the MLP model in the confusion matrix (Fig. 9) shows the strong classification performance for all the classes of beverage stains. There are minimal misclassifications between the spectrally similar classes of stains, which proves the efficiency of the proposed method using the ANOVA band selection and the MLP model. The training process of the model is further shown in Fig. 10, in which the validation loss decreases smoothly and the validation accuracy increases throughout the training process. This confirms the resilience of the suggested approach for hyperspectral stain classification

**Fig. 11:**
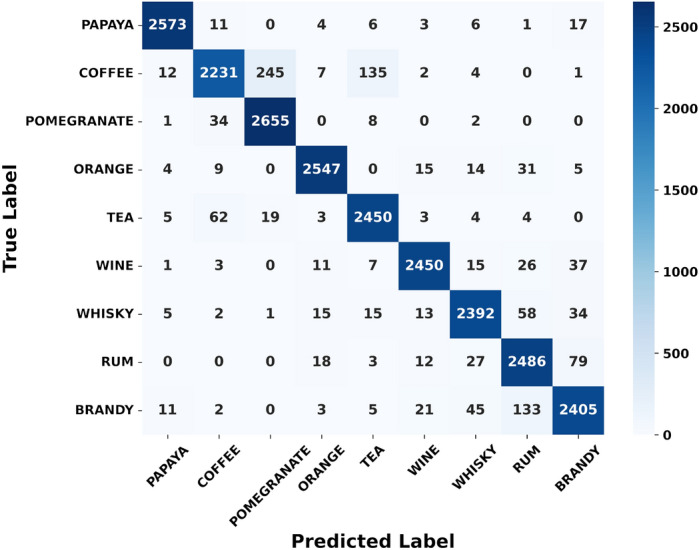
Confusion matrix of the 1D-CNN model for beverage stain classification.

**Fig. 12:**
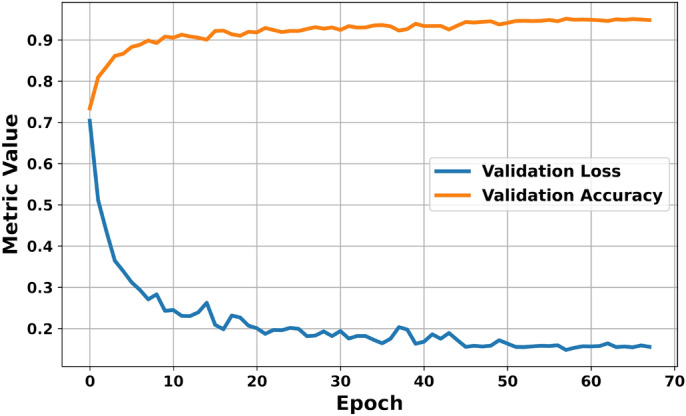
Validation loss and validation accuracy curves for the 1D-CNN model.

***1D-CNN model*** The 1D CNN model is successful in capturing local spectral dependencies present in the hyperspectral data. This is demonstrated through the confusion matrix, as shown in Fig. 11, which indicates high classification accuracy for all beverage stain classes, under the influence of a dominant diagonal, implying that the majority of instances have been correctly classified by the model. However, minor misclassifications have been observed between spectrally similar beverage types, such as Rum and Brandy, which indicates similar spectral characteristics between these two beverage types. The training behavior of the 1D CNN model is demonstrated through Fig. 12, which indicates that the validation loss is gradually decreasing, and validation accuracy is consistently increasing, implying stable learning and good generalization performance for hyperspectral stain classification.

**Fig. 13:**
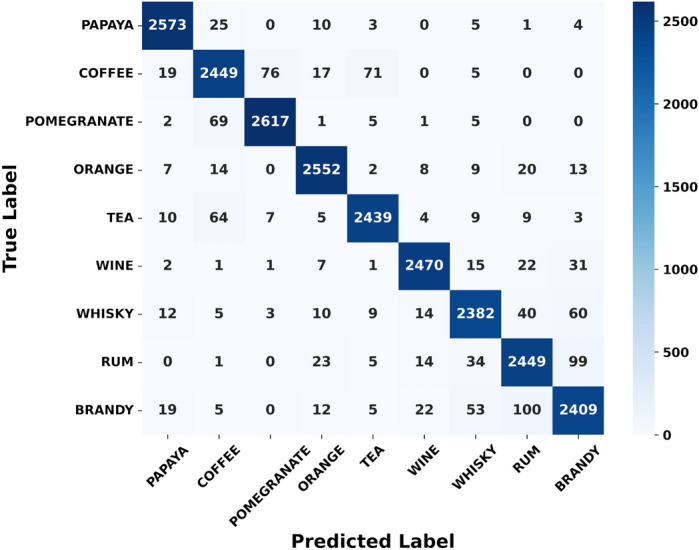
Confusion matrix of the LSTM model for beverage stain classification.

**Fig. 14:**
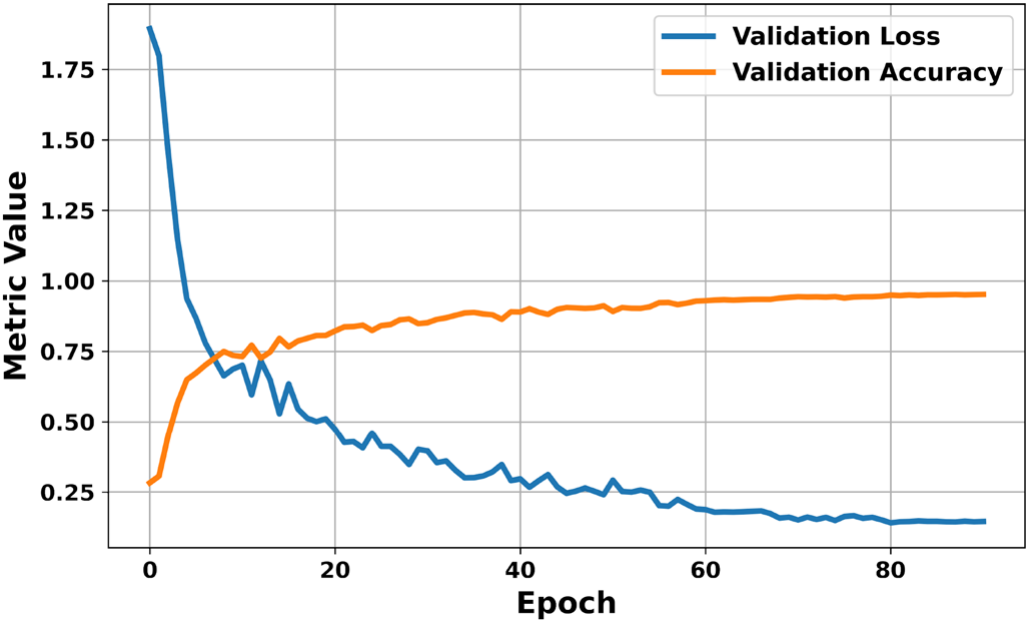
Validation loss and validation accuracy curves for the LSTM model.

***LSTM model*** The LSTM model utilizes its recurrent structure with gates to effectively tap into the long-range dependencies that are present in the spectral domain of the hyperspectral images. This is evident in the confusion matrix in Fig. 13, where the LSTM model demonstrates high performance in classifying the different classes of beverage stains, as shown by the high values along the diagonal. Although some level of confusion is observed between the Coffee and Pomegranate classes, this is not unexpected since these two classes share similar spectral signatures. In the learning behavior of the model in Fig. 14, the validation loss is shown to decrease while the validation accuracy increases.

**Fig. 15:**
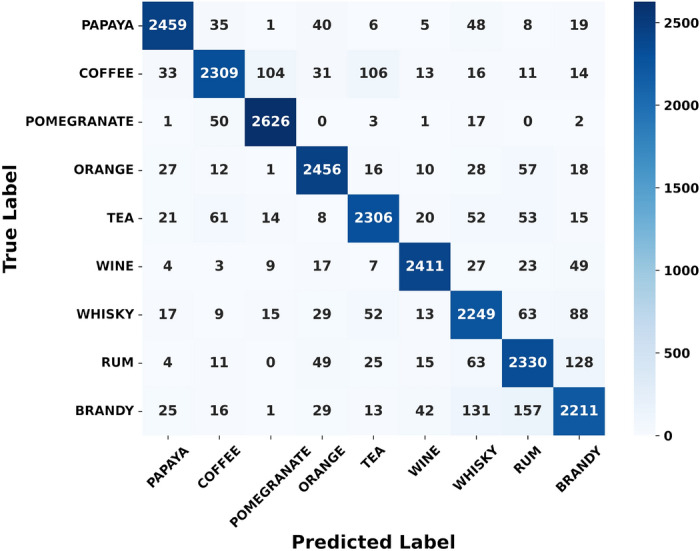
Confusion matrix of the CNN–LSTM hybrid model for beverage stain classification.

**Fig. 16:**
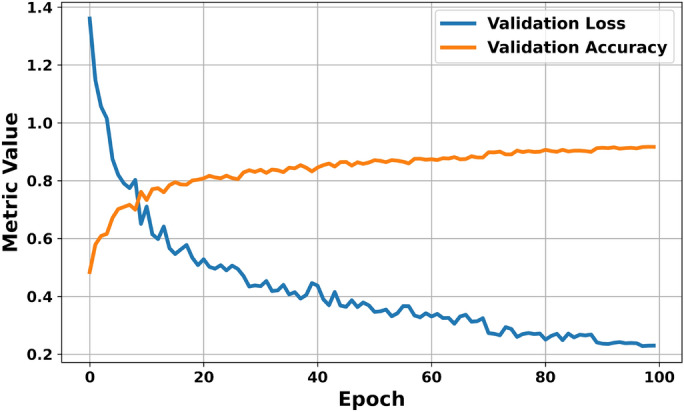
Validation loss and validation accuracy curves for the CNN–LSTM hybrid model.

***CNN–LSTM hybrid model*** The CNN-LSTM hybrid model uses convolutional layers for the extraction of local spectral features and recurrent layers for the modeling of sequential dependencies in hyperspectral data. As depicted in the confusion matrix in Fig. 15, the CNN-LSTM model exhibits high efficacy in the categorization of most beverage stains, as depicted in the diagonal of the matrix. However, the presence of off-diagonal values in the matrix indicates an increase in misclassifications compared to the other models. This is especially observed for classes of beverages with similar spectral signatures, such as the classes for Rum, Brandy, and Whisky. The training dynamics of the CNN-LSTM model are illustrated in Fig. 16, showing the decrease in the loss function for the validation set during the initial epochs, followed by fluctuations in the later stages of the optimization process. This indicates difficulties in optimization and the possibility of overfitting for deeper architectures using the limited hyperspectral data for training.

#### Stain-pair comparison

**Fig. 17:**
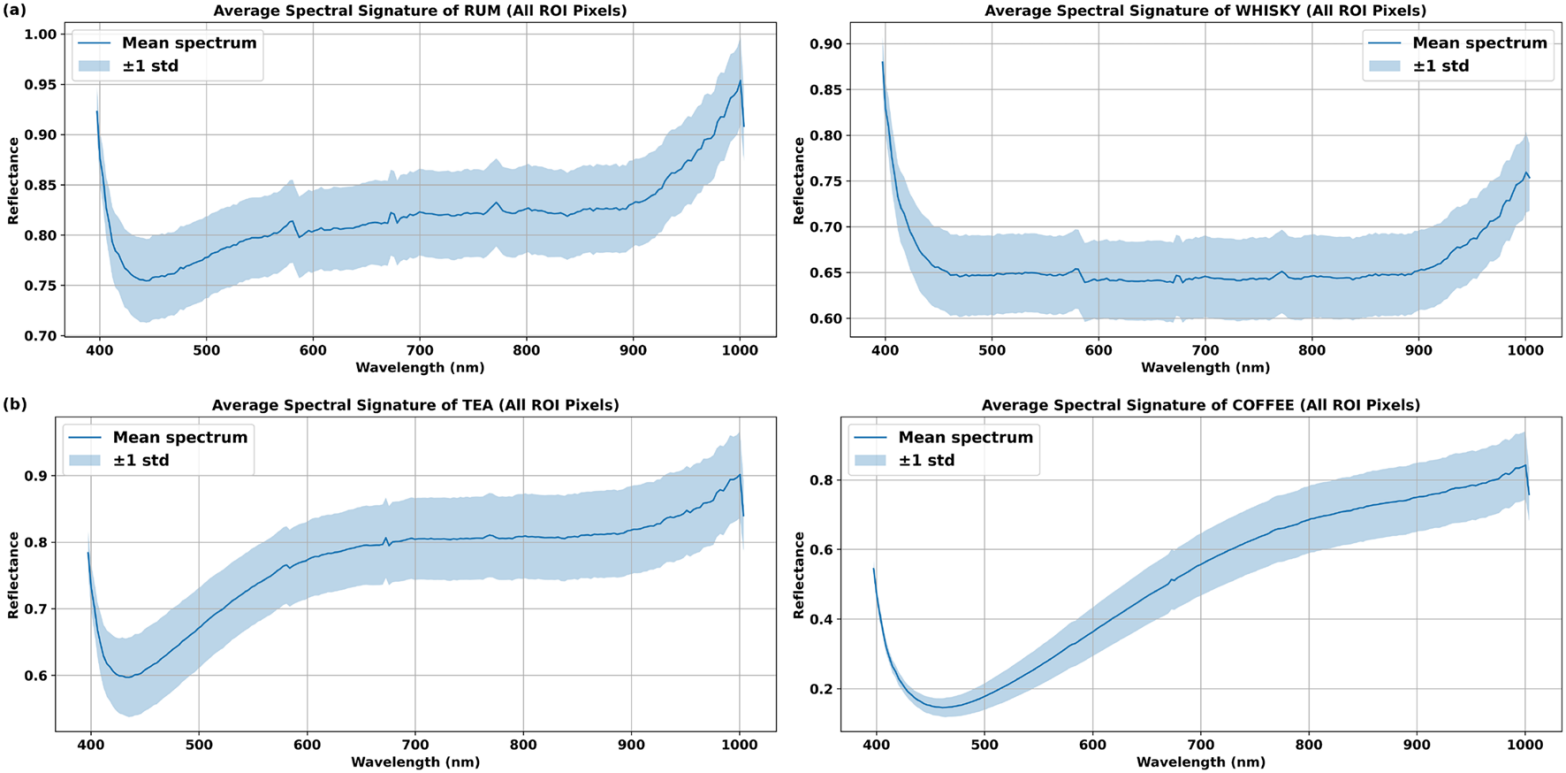
Spectral comparison of confusing stain pairs. (**a**) Rum vs Whisky (**b**) Tea vs Coffee.

To further validate the patterns of confusion that are being observed during the classification process, pairwise spectral comparison was conducted for certain stain pairs. Fig. 17 illustrates the spectral comparison of Coffee-Tea and Rum-Whisky. It is being observed that Coffee and Tea have similar spectral characteristics in the visible region (400–550 nm), which is causing a higher rate of misclassification. It is also being seen that Rum and Whisky are showing similar reflectance characteristics in the near-infrared region (750–900 nm), making it difficult to classify them. This validates the understanding that the difficulties being faced during the classification process are actually due to the spectral similarities of certain stain pairs.

### Inference and evaluation against traditional techniques

**Fig. 18:**
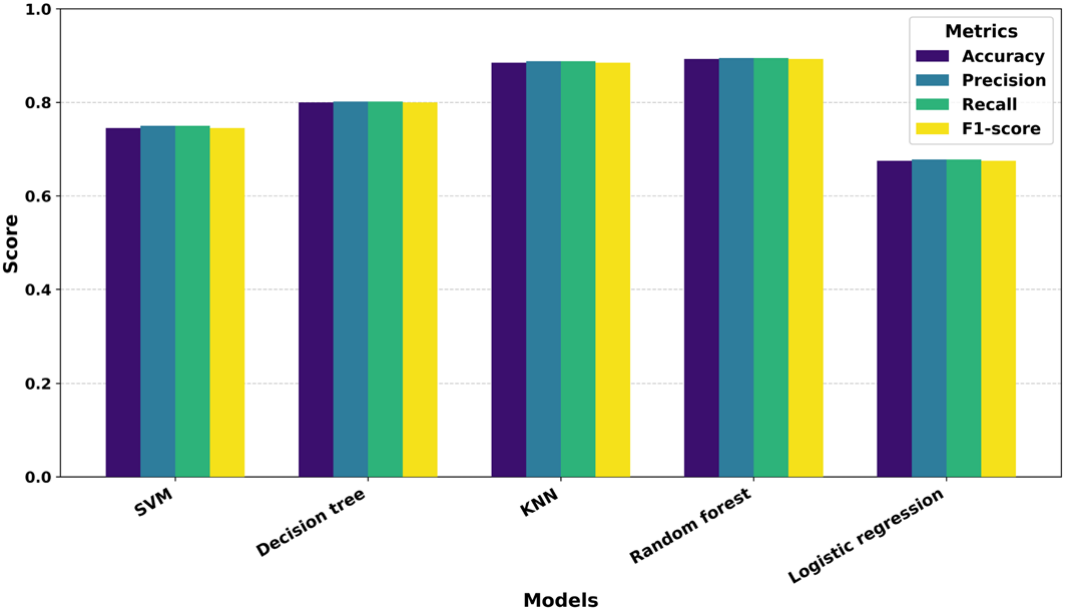
Comparison of several different ML algorithms.

**Fig. 19:**
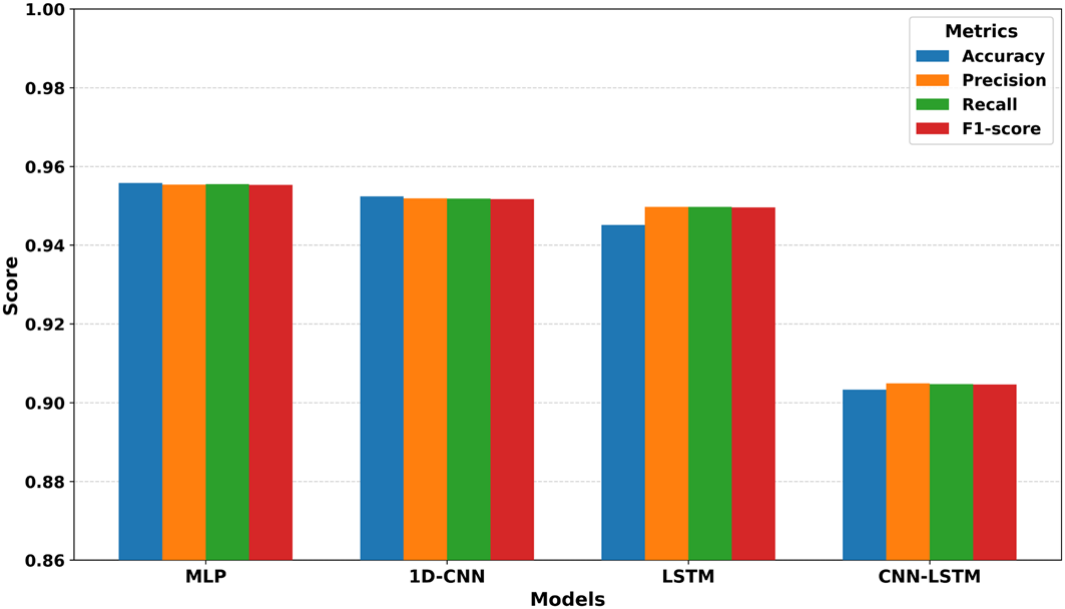
Comparative study of deep learning algorithms.

Deep learning models outperformed classical Machine Learning (ML) methods in distinguishing beverage stains from hyperspectral data. The performance of the classical ML algorithms, namely, Support Vector Machine, Decision Tree, *k*-Nearest Neighbors, Random Forest, and Logistic Regression, was reasonably good with the 162 ANOVA-selected spectral bands. However, these methods are based on hand-designed features, which cannot capture the complex nonlinear spectral correlations in hyperspectral data, as depicted in Fig. 18. These spectral-spatial dependencies are learned automatically by deep learning architectures, namely MLP, 1D-CNN, LSTM, and CNN-LSTM, as shown in Fig. 19. Among these techniques, the MLP provided the maximum accuracy, closely followed by 1D-CNN and LSTM. The CNN-LSTM hybrid showed good performance but with reduced accuracy because of increased model complexity. Generally, the deep learning models showed higher accuracy, adaptability, and scalability in performance compared to the traditional ML methods. They indeed proved to be effective in modeling hyperspectral spectral variability and classifying visually similar stains.

## Discussion

The proposed MLP model has attained an accuracy of 95.58%, which confirms that smaller neural network models are capable of learning discriminative spectral patterns when coupled with statistically informed band selection methods. The accuracy attained confirms that the selected spectral features are sufficiently informative for differentiation between visually similar beverage stains. The comparative analysis of the proposed 1D CNN and LSTM models has provided additional insight into the characteristics of the hyperspectral signatures, confirming the local and sequential patterns within the signatures. Although CNN models possess the ability to learn local patterns, and LSTM models have the capacity to learn sequential patterns, the best performance was attained by the MLP model, confirming that global spectral models and effective feature selection are sufficient for differentiation between chemically similar beverages such as rum and whisky. From a methodological point of view, the integration of statistical feature selection and deep learning minimizes spectral redundancy while retaining important wavelengths. This method enhances the efficiency and stability of the learning process, ensuring robust classification performance across beverage classes. From a forensic point of view, hyperspectral imaging technology holds tremendous promise for the automatic and non-destructive analysis of stains. This is because it is capable of carrying out contactless examination of stains while maintaining the integrity of evidence. This technology can be very useful in the field of forensic science because it is able to provide objective support in the identification of stains, especially when chemical reagents cannot be used.

### Comparison with existing forensic HSI studies

To the best of our knowledge, the only comprehensive forensic study specifically addressing beverage stain analysis using hyperspectral imaging is the work by Devassy and George (2021)^4^. Table 9 summarizes the methodological differences between their study and the proposed framework.

**Table 9:** Comparison with existing forensic hyperspectral beverage stain analysis studies.

Aspect	Devassy & George (2021)	Proposed Work
Dataset	12 beverages on paper towel substrates	9 beverage stains on tissue substrates
Band Selection Method	Volume Gradient Based Band Selection (VGBS)	ANOVA-based supervised band selection
Dimensionality Reduction	Convolutional Auto-Encoder (CAE)	Statistical feature selection (ANOVA)
Classification Model	Support Vector Machine (SVM)	MLP, 1D-CNN, LSTM, CNN-LSTM
Evaluation Strategy	Train–test split with cross-validation	Stratified train–validation– test split and 5-fold cross-validation
Reported Accuracy	93.30% test accuracy	$\sim$95–96% accuracy (MLP model)

The comparison indicates that the proposed framework improves classification accuracy while also providing a broader evaluation of deep learning architectures for hyperspectral forensic analysis.

### Limitations

There are a few limitations associated with the present work. First and foremost, the experiments were conducted on tissue paper substrates (white, pink, and brown) under controlled indoor illumination conditions. However, the performance on other relevant substrates such as cotton fabrics, denim, paper documents, human skin, etc., is not yet evaluated. Secondly, the work was conducted on single stains only. However, stains may overlap with each other during a crime scene. Overlapping stains may change the classification complexities. Thirdly, another limitation is that the temporal analysis was restricted to a 0–5 hour observation window. In real forensic scenarios, stains may remain on surfaces for significantly longer durations. Lastly, the cost associated with the forensic error was not evaluated. However, the cost associated with the FP and FN is not the same during a crime scene.

## Conclusion and future work

This research presents an HSI framework that integrates ANOVA-based band selection with deep learning for the classification of beverage stains in forensic investigations. The results demonstrate that spectral features extracted from hyperspectral data, combined with deep neural models, enable accurate and non-destructive recognition of visually similar stains on absorbent substrates. The MLP model outperformed all of the others in terms of classification accuracy, reaching 95.58%, outperforming 1D-CNN, LSTM, and CNN-LSTM architectures. These findings indicate that even relatively simple neural network models can effectively capture discriminative spectral characteristics from high-dimensional HSI data when appropriate feature selection and regularization strategies are applied. Overall, the proposed framework provides a reliable basis for rapid, contactless, and reproducible forensic stain analysis.

There are several avenues for further research in the proposed framework. In one such direction, group-based data partitioning at the stain instance level will be explored for a more rigorous assessment of model generalization performance. In addition, the proposed framework will be extended to include both spectral and spatial information by utilizing advanced architectures such as 3D CNN for spectral-spatial feature learning. Another direction for further research is to include temporal information for better representation learning in capturing stain aging effects over various time periods. Further study will also include model calibration analysis utilizing reliability diagrams and expected calibration error (ECE) to evaluate the confidence and reliability of predicted probability. Additionally, ensemble learning models based on CNN, LSTM, and Transformer models will be explored for better classification performance. The dataset will be expanded to include diverse beverage varieties, substrate materials, and various climatic conditions to enhance robustness in real-world forensic situations. These initiatives seek to establish an efficient and sophisticated decision support system for accurate stain detection and classification.

## Data Availability

The data used and/or generated during the current study are available from the corresponding author on reasonable request.
